# Dengue: 30 years of cases in an endemic area

**DOI:** 10.6061/clinics/2019/e675

**Published:** 2019-09-04

**Authors:** Daniela Cristina Sensato Monteiro, Natália Vasconcelos de Souza, Jane Cavalcante Amaral, Kaynan Bezerra de Lima, Fernanda Montenegro Carvalho de Araújo, Izabel Letícia Cavalcante Ramalho, Victor Emanuel Pessoa Martins, Jeová Keny Baima Colares, Luciano Pamplona de Góes Cavalcanti, Danielle Malta Lima

**Affiliations:** I Programa de Pos Graduacao em Biotecnologia, Universidade Estadual do Ceara, Universidade de Fortaleza, Fortaleza, CE, BR; II Universidade de Fortaleza, Fortaleza, CE, BR; III Laboratorio Central de Saude Publica do Ceara, Fortaleza, CE, BR; IV Universidade de Integracao Internacional da Lusofonia Afro Brazileira (UNILAB), Redencao, CE, BR; V Departamento de Saude Comunitaria, Universidade Federal do Ceara, Fortaleza, CE, BR

**Keywords:** Dengue, *Aedes Aegypti*, *Aedes Albopictus*, Virus

## Abstract

The present study aimed to review literature on studies of dengue cases conducted over 30 years in the state of Ceará.

Between November 2015 and January 2016, articles published in Portuguese and English in 7 databases were searched using keywords and a Boolean operator. A total of 191 articles were identified in the databases; 133 were excluded according to the exclusion criteria, and 58 were included in the study.

Of the 58 articles analyzed, 6 reported data from Brazil; including the Northeast region and the state of Ceará; 41 reported data for only the city of Fortaleza; 7 reported data for the state of Ceará; 4 reported data for cities in the interior of the state; and 3 included only children. The studies adopted different approaches and focused on different aspects of the disease. Study outcomes included the identification of serological, epidemiological, clinical, and laboratory characteristics; potential larvicides and biological predators of mosquitoes; potential antiviral agents; vector density characteristics; and educational dengue prevention and control strategies. Additionally, one vaccine trial was included.

Although studies on dengue in the state of Ceará are scarce, they are encompassing, including several lines of research, and the number of studies and reports on dengue in the state of Ceará continues to increase.

## INTRODUCTION

The first dengue epidemic in Brazil is believed to have occurred between 1846 and 1853 in the cities of São Paulo and Rio de Janeiro (RJ) 1,2. At that time, the disease was known by other names, such as polka (a fashionable dance at the time), polka fever, and break-bone fever 3. However, dengue cases were first reported in medical literature in 1916 in the city of São Paulo and in 1923 in Niterói 1,2_._ The first epidemic documented from a clinical and laboratory viewpoint occurred in late 1981 and early 1982 in Boa Vista, Roraima; this epidemic was caused by the dengue-1 (DENV-1) and dengue-4 (DENV-4) serotypes 4. In 1955, Brazil succeeded in eradicating *Aedes aegypti (A. aegypti)*, eliminating the last mosquito source on the 2nd of April in a rural area in the municipality of Santa Terezinha, Bahia 5.

The first cases of *A. aegypti* reinfestation following eradication occurred in 1967 in Belém (Pará) and in 1968 in São Luiz (Maranhão); the source populations were finally eliminated in 1973. *A. aegypti* was again detected in 1976 in Salvador (Bahia) and in 1977 in RJ and then spread to other states 6,7. In 1986, a dengue epidemic (DENV-1 serotype) circulated in the state of RJ and quickly reached Northeast Brazil 7,8. From 1986 to 1990, dengue epidemics were limited to some Brazilian states in the Southeast (RJ, São Paulo and Minas Gerais) and Northeast regions (Alagoas, Bahia, Ceará and Pernambuco) 9.

*A. aegypti* was reintroduced in the Ceará cities of Aquiraz, Beberibe and Fortaleza in the mid-1980s 10. The first dengue cases occurred in August 1986, originating from tourists from RJ, where a dengue epidemic (DENV-1) was occurring, who were visiting the cities of Fortaleza and Canoa Quebrada 11.

For the dengue cases reported in the state of Ceará over 30 years, seven epidemics were recorded (1987, 1994, 2001, 2008, 2011, 2012 and 2015) 12,13. Ceará has 184 municipalities, 167 of which reported DENV transmission in 2015. From 1986 until 2016, 302,015 dengue cases were reported in the state 14. Dengue has therefore been endemic to Ceará for 30 years, resulting in high incidence rates caused by four circulating serotypes. The present study aimed to review literature on studies involving dengue cases in the state of Ceará conducted during these 30 years.

## METHODOLOGY

**Figure 1:**
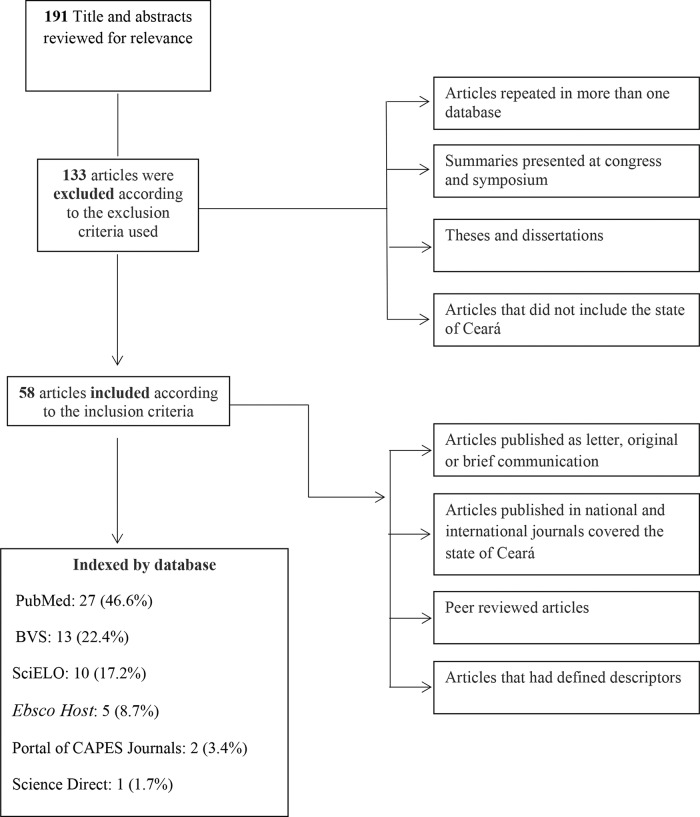
Flow chart - Selection of articles. Notes: Biblioteca virtual em saúde (BVS).

Between November 2015 and January 2016, articles in the Portal of CAPES Journals, Biblioteca virtual em saúde (BVS), Ebsco Host, Scientific Electronic Library Online (SciELO), PubMed, Science Direct *and Google Scholar* databases were searched. Scientific reports about dengue in Ceará from 1986 until 2016 were identified. The search was performed in both Portuguese and English using the Boolean operator “and” and the following keywords: “Dengue and Ceará”, “Dengue and Fortaleza”, “Dengue virus and Ceará”, “Dengue virus and Fortaleza”, “Dengue virus Ceará” and “Dengue virus Fortaleza”. To organize the information contained in the scientific articles identified with the descriptors, the articles were fully read, and the objectives, study type, location, period, methods, importance and results were identified. Published scientific reports such as letters, original articles and brief communications to national and international journals were included in the search according to the descriptors and peer reviewed. After preanalysis, scientific literature identified in more than one database that was accounted for as a single work, congress abstracts, theses, dissertations and articles that included no data for the state of Ceará were excluded. A total of 191 articles were identified in the databases; 133 articles were excluded according to the exclusion criteria, and 58 articles were included in the study (Figure 1).

## RESULTS AND DISCUSSION

Of the 7 databases analyzed, only Google Scholar did not contain articles related to the descriptors of the present work.

Of the 58 reports analyzed, 6 (10.3%) reported data from Brazil, including the Northeast region and the state of Ceará; 41 (70.6%) reported data for only the city of Fortaleza; seven (12%) reported data for the state of Ceará; and four (6.8%) reported data for cities in the interior of the state (one in Icaraí (Caucaia); one in Juazeiro do Norte, Crato and Barbalha; one in Tauá and one in Pacoti) (Tables 1 and 2). Of the reports analyzed, 39 (67.2%) included only adults, 16 (27.5%) included children and adults, and 3 (5.2%) included only children 15-17.

### Epidemiological aspects

A literature study about dengue and the control of *A. aegypti* in Ceará, based on epidemiological bulletins, found that five dengue epidemics, with high incidence rates, occurred in Ceará between 1986 and 2011, and children were most affected from 2008 to 2010. Annually, during the studied period, an average of 120 municipalities reported infestation with *A. aegypti,* and 84 reported dengue transmission 18.

In 2014, the city of Fortaleza was a World Cup host city, and a study was performed to investigate the potential for a dengue epidemic in the 12 World Cup host cities 19. Real-time seasonal climate forecasts were performed based on several international sources, and epidemiological predictions for dengue in Brazil were analyzed. The results showed reduced risks of dengue in the cities of Brasília, Cuiabá, Porto Alegre and São Paulo. Some cities were considered to have moderate risk (Belo Horizonte, Salvador and Manaus), and some cities, such as Recife, Natal and Fortaleza, located in Northeast Brazil, were considered to have high risk 19.

### Clinical and laboratory aspects

For the period analyzed (1986-2016), we selected manuscripts that described the clinical and laboratory aspects of the disease in Ceará (Table 1).

### Larvicides and biological predators/antiviral drugs/vectors

Larvicides and biological predators are used as methods to fight the spread of mosquitoes. In the present review, following stratification of the 58 selected reports, 13 (22.4%) were found to address the use of larvicides and biological predators against *A. aegypti* 40-52 (Table 2). Other studies examined the vectors *A. aegypti* and *Aedes albopictus* (*A. albopictus)* 53-58 or investigated antiviral drugs against dengue 59,60. The main findings of these studies are listed below (Table 2).

### Educational actions

After stratification, two reports about educational actions were found. The first was performed in Icaraí (Caucaia) and concerned dengue prevention and control actions, evaluating the limitations and difficulties found. The seventeen individuals who participated in the study were distributed into three groups: eight were users of a Basic Health Unit, four were endemic disease control agents, and five were health care professionals. Data were collected by participant observation and professional/user interactions that occurred throughout the study. The study concluded that actions that strengthened the power and responsibility of individuals over their own history and their local citizenship were needed 61. The second report was a case study involving six blocks, with each block corresponding to 40 houses in Fortaleza. The aim of the study was to evaluate the implementation of an intervention strategy to decrease the amount of *A. aegypti.* Fortaleza is divided into six administrative regions (SERs), and in this study, one block in each of the six SERs was selected to understand the ecological, biological, and social complexity and diversity in Fortaleza. The results showed a complex interaction between socioenvironmental inequalities and dengue control. The ethnographic data and interviews of those in the studied SERs reflected the environmental and socioeconomic complexity of a large city in a developing country such as Brazil, which has struggled with the serious public health problem of dengue 62.

Two reports concerning risk factors associated with dengue were included. One case-control study analyzed low-income housing development on the coast of Fortaleza with the aim of increasing the efficacy of control measures against dengue during a dengue outbreak. A total of 211 households were investigated using a questionnaire to collect socioeconomic, behavioral, and environmental risk data for cases and controls. Cases were defined according to the national guidelines for the control of dengue based on the detection of dengue IgM antibodies; 34 cases and 34 controls were investigated. An analysis of the epidemic in a low-income housing development showed the presence of several known risk factors because human behavioral components are difficult to manipulate. However, other factors, such as the frequency of visits by vector control agents, could be solved by making simple changes within municipal services 63.

Another study evaluated the implementation of an intervention strategy to decrease the amount of *A. aegypti* in 2012 and 2013. Participants were divided into 10 intervention groups and 10 control groups, and activities such as workshops, cleaning campaigns, and student and older inhabitant mobilization were conducted. Differences in social participation, commitment and leadership were observed between the groups, and a higher efficacy was observed in the intervention group than in the control group. Social participation and environmental management are viable and promising alternatives to vector measures for dengue vector control 64.

## CONCLUSION

Dengue remains a serious public health problem and constitutes a threat to the Brazilian population. The tropical climate of Brazil favors the proliferation of dengue and the four serotypes (DENV-1 to DENV-4) that circulate in Brazil. Vector control is still the most effective means of protection against the spread of the disease but remains ineffective against the numerous epidemics occurring every year in Brazilian cities. Serological diagnostic tests also need to be improved because cross-reactions with other arboviruses often occur.

From the present review, we conclude that although studies on dengue in the state of Ceará are scarce, they are encompassing, including several lines of research, and the number of studies and reports on dengue in the state of Ceará continues to increase.

## AUTHOR CONTRIBUTIONS

Monteiro DCS contributed to the study design, data collection, analyzed and interpreted data. Cavalcanti LPG and Colares JKB assisted with the design of the study survey, data interpretation and manuscript reviews. Araújo FMC assisted with data interpretation, and manuscript writing and review. Martins VEP and Ramalho ILC reviewed the manuscript. Souza NV, Amaral JC and Lima KB participated in the conceptualization of the study, data collection and manuscript reviews. Lima DM conceived the study, participated in the study design, coordination, and reviewed the manuscript. All authors read and approved the final version of the manuscript.

## Figures and Tables

**Table 1:** Main characteristics of the stratified studies with clinical and laboratory characteristics.

Clinical and laboratory characteristics of the studies				
Date	Study Design	Objective	Main Results	Reference
1995	Descriptive	To evaluate the cases of hemorrhagic dengue fever in the city of Fortaleza in 1994.	Of the 27,033 confirmed cases in the state and 19,306 in Fortaleza, 178 were suspected to be hemorrhagic dengue, which was confirmed in 26 patients, 11 of which had shock.	20
1998	Descriptive	To conduct a seroepidemiological survey to evaluate the prevalence of dengue after the dengue epidemic (1994) in Fortaleza, Ceará.	A total of 1,341 serum samples were evaluated, and of these, 44% were dengue positive and 56% were dengue negative.	21
1998	Retrospective	To detect the presence of the dengue virus before the 1994 epidemic.	Serological diagnosis was confirmed in 24.4% of the participants by detecting anti-IgM antibodies and confirming dengue infections in Fortaleza in November 1993, six months before the epidemic.	22
2006	Descriptive	To report a case of DENV-2 and DENV-3 coinfection.	The first case of simultaneous infection by DENV-2 and DENV-3 in Brazil was documented.	23
2009	Descriptive	To report a case of aplastic anemia induced by dengue virus infection.	Dengue can induce aplastic anemia by directly invading the bone marrow. This rare complication must be identified early. Immunosuppressive therapy may induce complete remission.	24
2010	Descriptive	To report cases of optic neuritis after dengue virus infection.	Two cases of bilateral neuritis after dengue virus infection were described.	25
2011	Retrospective	To analyze the main pain-related complaints in patients with dengue.	Of the 94.8% patients who presented with at least one pain complaint, patients diagnosed with DHF (40.1%) had more painful symptoms than those diagnosed with CD, and the main complaints were headache (79.9%) followed by myalgia (78.6%).	26
2011	Descriptive	To detect cases of hantavirus in patients with clinical suspicion of dengue.	One patient was IgM-positive for hantavirus and two were IgG-positive for hantavirus. Therefore, it is important to improve epidemiological surveillance for hantavirus in the state of Ceará.	27
2012	Retrospective	To correlate laboratory tests during the progression of dengue fever with symptoms to predict the severity of the disease.	In patients with CD, thrombocytopenia and elevated transaminase levels were observed; in those with DHF, the thrombocytopenia and elevated transaminase levels were similar to those in patients with SD, while the hemoconcentration was not. The results can be used as markers for more severe forms of the disease.	28
2012	Descriptive	To determine the frequency of CNS infection by the dengue virus in individuals with fatal outcomes.	Clinical manifestations and positive laboratory results in CSF may indicate the presence of DENV and lead to the consideration of CNS invasion in fatal cases.	29
2012	Retrospective	To evaluate the prevalence of dengue in patients with suspected viral meningitis.	Dengue should be suspected in patients in endemic areas with neurological manifestations, and appropriate treatment should be adopted to avoid fatality.	30
2012	Descriptive	To report a fatal case of coinfection with severe dengue and melioidosis.	Melioidosis should be considered among differential diagnoses in endemic disease areas.	31
2013	Retrospective	To report myocarditis due to dengue, which is rarely diagnosed.	Myocarditis caused by DENV occurred in four confirmed cases. Therefore, there is a need to assess cardiac function in all patients with acute dengue who may benefit from therapy to prevent death from heart disease.	32
2013	Retrospective	To describe the clinical spectrum of dengue in children and adolescents.	The main signs and symptoms of dengue are fever, abdominal pain, and vomiting. A unique clinical profile, including gastrointestinal symptoms and hepatic involvement, was obvious.	15
2014	Descriptive	To report the first case of dengue fever in an indigenous child who died.	With positive immunohistochemical results, the case was confirmed as severe dengue. Doctors should consider dengue as a diagnostic hypothesis among the indigenous populations in Brazil.	17
2014	Retrospective	To evaluate the new WHO 2009 classification of dengue.	The revised classification for detecting severe clinical manifestations has allowed better detection in patients with SD and can thus reduce fatalities.	33
2015	Descriptive	To investigate the hypothesis that some specific comorbidities increase the likelihood of DF progressing to DHF in adults.	The progression to DHF was associated with hypertension and skin allergy. Therefore, these patients should remain in healthcare facilities to monitor progression.	34
2015	Descriptive	To describe clinical manifestations and renal involvement in cases of dengue in renal transplant patients.	Of the renal transplant recipients, 10 were diagnosed with dengue, 5 were hospitalized, 4 developed DHF, and none died.	35
2015	Descriptive	To diagnose possible cases of leptospirosis in dengue-negative patients in samples from 2008, 2010, and 2012.	Patients with suspected dengue and those negative for dengue were tested for leptospirosis; 10.8% (2008), 19.2% (2010), and 30.8% (2012) were confirmed to have leptospirosis. The authors estimate that 20% of dengue cases may actually be cases of leptospirosis in endemic disease areas.	36
2015	Descriptive	To identify acute febrile episodes to describe the density of incidence, efficacy, and seroprevalence.	This study captured and monitored patients with dengue who were selected to participate in a phase III dengue vaccine trial. Of 235 children with acute febrile episodes, 50 (21.3%) were considered likely to have dengue, and 18 (7.7%) had virologically confirmed dengue.	16
2015	Descriptive	To analyze cases of severe dengue in the early postoperative period of renal transplantation.	After performing renal transplantation, two severe cases of dengue were reported. The authors report the importance of screening for dengue before transplantation in endemic disease areas.	37
2015	Descriptive	To investigate leptospirosis in patients with clinical suspicion of dengue.	Of the patients analyzed, 48 (55.8%) were positive for dengue in at least one of the tests, and 5 (7.35%) were positive for leptospirosis.	38
2016	Retrospective	To report the detection of undeclared dengue deaths.	Ninety dengue deaths were detected that were not suspected during disease progression. The authors suggest the need to improve primary health care to identify cases of fatal dengue and thus prevent death.	39

Notes: DENV: dengue virus, CD: classic dengue fever, SD: severe dengue, DHF: dengue hemorrhagic fever, CSF: cerebrospinal fluid, CNS: central nervous system, IgM: immunoglobulin M, IgG: immunoglobulin G; WHO: World Health Organization.

**Table 2:** Main results of published studies on biological predators, larvicides, antiviral drugs and vectors.

Biological Predators, Larvicides, Antiviral Drugs, and Vectors				
Study Design	References	Fish Species	Objective	Main Results
**Experimental**	40	*Betta splendens (B. splendens), Poecilia reticulata*	To evaluate the oviposition behavior of *A. aegypti* in containers containing *B. splendens* and *P. reticulata*.	*B. splendens* was better at controlling *Aedes* mosquitoes than *P. reticulata* and can be used to prevent *A. aegypti* females from depositing eggs in water containers.
**Experimental**	41	*B. splendens*	To estimate the survival of *B. splendens* in domestic containers and their efficacy in controlling premature stages of *A. aegypti* compared to the larvicide *Bacillus thuringiensis israelensis* (Bti).	*B. splendens* may be suitable for the biological control of *A. aegypti* in large domestic water containers, but measures should be taken to ensure prolonged survival and the presence of fish in the containers.
**Experimental**	42	*B. splendens, Poecilia sphenops, Trichogaster trichopterus*, and *Astyanax fasciatus*	To evaluate the competence of fish on the predation of *A. aegypti* larvae under laboratory conditions.	Females and males of *T. trichopterus* and *A. fasciatus* and females of *B. splenden*s and *P. sphenops* were the most competent fish for *A. aegypti* larvae predation.
**Experimental**	43	*B. splendens*	To evaluate the use of larvivorous fish in cement tanks as a form of biological control for *A. aegypti* larvae.	*B. splendens* showed potential for biological control in cement tanks, reducing the infestation by 320-fold in this type of container.
**Experimental**	44	*B. splendens, T. trichopterus,* and *P. reticulata*	To evaluate the survival of predator fish larvae in water with larvicides used to control *Aedes*.	*B. splendens* presented the lowest mortality rate, and the combined use of predatory and larvicidal fish in large water tanks is feasible.
		**Plant Species Used**	**Native Plants As Larvicides**	
**Experimental**	45	Constituents of the natural liquids of the cashew nutshell: anacardic acid, cardol, and cardanol	To evaluate the antioxidant and larvicidal actions of the components anacardic acid, cardanol, and cardol.	The three components were shown to be promising agents to control *A. aegypti* and function as antioxidants, acetylcholinesterase inhibitors, and *A. aegypti* larvicides.
**Experimental**	46	Cashew nut shell: sodium anacardate	To evaluate the insecticidal activity of sodium anacardate isolated from cashew nut shell liquid against the eggs and pupae of *A. aegypti.*	Sodium anacardate presented toxicity against *A. aegypti* eggs, larvae, and pupae and may be a viable, low-cost alternative to control *Aedes.*
		**Type of Oil**	**Essential Oils**	
**Experimental**	47	*Alpinia zerumbet, Citrus limonia, Citrus sinensis, S. jambolana, Ocimum americanum, Ocimum gratissimum, Hyptis suaveolens*	To evaluate the larvicidal activity of nine plants found in Northeast Brazil against *A. Aegypti* larvae.	*O. americanum* and *O. gratissimum* have LC_50_ values of 67 ppm and 60 ppm, respectively, and the authors suggest a beneficial use of these essential oils for controlling *A. aegypti.*
**Experimental**	48	*Capraria biflora*	To evaluate the larvicidal activity of *C. biflora* against *A. aegypti.*	*C. biflora* oil contains 14 essential oil constituents and shows good larvicidal activity against *A. aegypti.*
**Experimental**	49	Leaves of *Hyptis martiusii* Benth	To evaluate the insecticidal activity of the chemical components of the volatile oils of *H. martiusii* Benth.	Twenty-six compounds, representing 93.2% of the leaf essential oils, were characterized, and the leaf essential oil and 1,8-cineole showed an insecticidal effect against *A. aegypti* larvae.
**Experimental**	50	Seed extract of *Myracrodruon urundeuva*	To isolate *m*-pentadecadienyl-phenol from *M. urundeuva* seeds and test its activity in three life stages of *A. aegypti* to elucidate its mode of insecticidal action.	*m*-Pentadecadienyl-phenol was shown to be a potent larvicide, with inhibitory activity on pupae and in the egg incubation phase.
		**Organophosphate**	**Chemicals**	
**Experimental**	51	Temephos	To evaluate the susceptibility of *A. aegypti* eggs and larvae to the organophosphate temephos.	Resistance to temephos was observed, demonstrating that populations of *A. aegypti* are under strong temephos selection pressure, compromising efficacy.
**Experimental**	52	Temephos and the pyrethroid cypermethrin	To describe resistance to temephos and the pyrethroid cypermethrin in three populations and to use biochemical and molecular assays to characterize resistance mechanisms.	Two populations of *A. aegypti* were under strong temephos selection pressure, compromising the efficacy of this organophosphate, and resistance to cypermethrin was observed in two of the three populations studied.
		**Mosquito**	**Vector/Mosquito**	
**Experimental**	53	*A. albopictus*	To publish the first record of *A. albopictus* in an urban area in the city of Fortaleza, state of Ceará.	Thirteen specimens of *A. albopictus* were identified (all female), and their introduction into Fortaleza was favored by the migration of people from other regions.
**Descriptive**	54	*A. aegypti* and *A. albopictus*	To identify the areas of occurrence, breeding sites, and associations with *A. aegypti* and other Culicidae.	The absence of one of the species at the breeding sites increased infestation by the other species, and breeding sites not infested by *A. albopictus* had twice the prevalence of *A. aegypti*.
**Experimental**	55	*A. aegypti*	To evaluate the daily survival probability of *A. aegypti.*	Survival analyses indicated that the mortality of *A. aegypti* varied with the age of the mosquitoes and with the local environment.
**Experimental**	56	*A. aegypti*	To estimate the variability of the survival rate of *A. aegypti* and determine how the climate is related to this variation.	The mortality of mosquitoes varies with age as well as with environmental and meteorological conditions. The climate in Fortaleza may impact the mortality of older mosquitoes that are potentially better pathogen vectors.
**Experimental**	57	*A. aegypti* and *A. albopictus*	To analyze the probability of vertical dengue virus transmission in *A. aegypti* and *A. albopictus* mosquitoes in the city of Fortaleza.	The vertical transmission of dengue virus in populations of *A. aegypti* and *A. albopictus* was found to be significant in the urban area of Fortaleza.
**Experimental**	58	*A. aegypti* and *A. albopictus*	To compare the incidence of dengue fever in relation to the rainfall in the city of Fortaleza since 1986.	The proportion of houses infested with mosquito vectors correlated inversely with the intensity of antimosquito interventions, and the vector population developed independently of rainfall.
		**Plant Species Used**	**Antiviral Drugs**	
**Experimental**	59	*Spondias tuberosa* and *Spondias mombin*	To evaluate new antiviral agents for controlling dengue virus. *Spondias* spp. extracts against DENV-2 were evaluated in C6/36 cells *in vitro*.	The main phenolic components rutin and quercetin showed substantial potential against dengue virus.
**Experimental**	60	Seeds of *Dimorphandra gardneriana*, *Adenanthera pavonina* and *Caesalpinia ferrea*.	To verify whether *D. gardneriana, A. pavonina*, and *C. ferrea* can be used as phytotherapy for dengue.	This was the first study to evaluate the antioxidant and antiviral effects of sulphated galactomannans against DENV-2. The results are promising and suggest that they act early during viral infection.

Notes: LC_50_: lethal concentration 50; DENV-2: dengue virus 2.
